# Application of Liquid Chromatography–Mass Spectrometry-Based Untargeted Metabolomics to Reveal Metabolites Related to Antioxidant Activity in Buckwheat Honey

**DOI:** 10.3390/molecules30102198

**Published:** 2025-05-17

**Authors:** Emilia Pogoda, Piotr Marek Kuś

**Affiliations:** Department of Pharmacognosy and Herbal Medicines, Faculty of Pharmacy, Wroclaw Medical University, ul. Borowska 211a, 50-556 Wrocław, Poland; emilia.pogoda@student.umw.edu.pl

**Keywords:** buckwheat honey, antioxidant activity, untargeted metabolomics, LC-MS, amadori compounds

## Abstract

Buckwheat honey is known for its high antioxidant activity, yet the compounds responsible for this effect have not been fully identified. This study used LC-MS-based untargeted metabolomics to investigate the metabolite profile of buckwheat honey and its relationship with antioxidant activity and total phenolic content, assessed by DPPH, FRAP, and Folin–Ciocalteu assays. A key objective was also to optimize data preprocessing parameters to improve the accuracy and robustness of metabolomic analyses. Multivariate analyses (PCA, OPLS-DA) effectively differentiated honey samples with high and low antioxidant potential. A total of 43 features were associated with increased antioxidant activity and about 30 compounds, including organic acids, free amino acids, and Amadori compounds—early Maillard reaction products—were identified. The amounts of most of these compounds exhibited strong positive correlation (r > 0.8) with measured antioxidant potential. These findings suggest that, in addition to polyphenols, other compound classes such as melanoidin precursors known as transition metal chelators significantly contribute to the antioxidant properties of buckwheat honey. This approach provides valuable insight into the bioactive composition of honey and supports the identification of potential antioxidant markers.

## 1. Introduction

Honey is a natural bee product that has been valued for centuries for its unique tastes and its health properties, which have been documented in many scientific studies. Its anti-inflammatory, antibacterial, and antioxidant effects are the main areas of interest in biomedical research [1,2,3]. Among the various types of honey, buckwheat honey obtained from the nectar of buckwheat flowers (*Fagopyrum esculentum* Moench) stands out for its special antioxidant activity. This effect is attributed to the high content of phenolic compounds such as flavonoids, phenolic acids, and their derivatives, which can neutralize free radicals [3]. This action is important in mitigating oxidative stress, a factor contributing to many diseases, including cancer, heart disease, and neurodegenerative diseases [1,4,5]. In addition, the mechanisms of action of these compounds include their ability to chelate metals, modify signaling pathways, and affect the activity of antioxidant enzymes [3,6]. However, recent studies suggest that the antioxidant activity of buckwheat honey may also be influenced by the presence of high molecular weight melanoidins, which are formed during the Maillard reaction. These melanoidins are recognized as major components responsible for the radical-scavenging capacity of both unheated and heat-treated honeys [7,8]. Despite the wide interest in this topic, the mechanisms responsible for the antioxidant activity of buckwheat honey have not yet been fully explained, probably due to the complexity of its composition and the various targeted methods used.

Advances in technology have directed researchers’ attention to untargeted assays to better characterize the substances being tested. In recent years, metabolomics has gained popularity in honey research—a field of science dealing with the analysis of metabolites in living organisms using advanced chromatographic and spectrometric techniques. This approach allows for a comprehensive analysis of the entire metabolome, and thanks to methods such as liquid chromatography–mass spectrometry (LC-MS), it is possible to perform untargeted analyses that allow the identification of a wide range of chemical compounds present in honey samples [9,10]. However, these studies are still challenging, mainly due to the huge amount of data generated, which require advanced analytical and algorithmic methods for their proper processing [11,12]. 

So far, metabolomics has been used in honey studies mainly in the context of distinguishing its varieties, determining the origin (botanical, geographical, etc.) of honey, its maturity, spoilage, as well as its bioactive potential, including antioxidant activity [13]. Shamsudin et al. applied metabolomic techniques such as gas chromatography–mass spectrometry (GC-MS) and liquid chromatography–quadrupole time of flight–mass spectrometry (LC-QTOF-MS) to identify metabolites related to antioxidant activity in stingless bee honey [9]. In the context of buckwheat honey, non-targeted methods have also been used, for example, to compare it with manuka honey, which has similar properties [14], or to classify buckwheat honeys from Kazakhstan based on their biochemical components and bioactive properties using a chemometric approach [15]. Both of these studies indicated compounds that may be responsible for the antioxidant activity of buckwheat honey. However, there is a need for further exploration of these issues, especially in relation to the comparison of buckwheat honey with other honeys with lower antioxidant activity. To the best of our knowledge, there is still a lack of comprehensive analyses that would clearly indicate the metabolites responsible for this specific property of honey. Most analyses focus on polyphenols, but as previously mentioned, recent studies highlight the importance of other compounds such as melanoidins in the context of the antioxidant properties of honey [8,16,17].

This study aims to optimize the methodology of metabolomics studies focusing on honey, paying attention to the preliminary steps in data analysis that can significantly affect the results. In addition, the study aims to identify metabolites associated with the antioxidant activity of buckwheat honey using LC-MS techniques, which will allow a better understanding of the mechanisms responsible for these properties and to compare them with honeys with much lower activity. By using an untargeted approach, this study has the potential to provide new information on the molecular basis of honey antioxidant activity and also contribute to the development of more precise methods for detecting bioactive compounds in different types of honeys.

## 2. Results and Discussion

### 2.1. Optimization of Data Processing Parameters

In untargeted metabolomics, the primary challenge lies in the handling and extraction of meaningful information from vast amounts of raw data. The complexity of the datasets generated, particularly through techniques like LC-MS, necessitates effective strategies to reduce data redundancy and noise while preserving critical information. The first essential step in metabolomic studies is the identification and selection of relevant signals from raw data, which involves reducing superfluous information and generating a clean dataset for subsequent analysis [11]. Data preprocessing plays a crucial role in metabolomic analyses, as it directly influences the reliability and quality of the data used in downstream statistical models. It encompasses a series of steps aimed at cleaning the data by removing noise, correcting for instrumental variability, and standardizing the data for consistency across samples. These steps include baseline correction, noise filtering, and normalization, all of which ensure that the data are free from systematic errors or biases that could skew the results [12,18,19]. Also, the choice of ionization mode can affect sensitivity or data loss, as some compounds do not ionize under given conditions [20,21]. Proper preprocessing is vital to avoid the loss of valuable metabolic signals, which could significantly impact the subsequent analysis [22]. For the current study, raw data obtained through LC-MS analysis were pre-processed using MetaboScape^®^ 2021b (Bruker, Billerica, MA, USA), which provided tools for aligning and deconvolution of the data. To investigate the effects of data processing parameters, multiple matrices were generated by altering key parameters such as different chromatographic separation, ionization mode, inclusion of quality control (QC) samples, batch correction, and different intensity thresholds. Specifically, 20 distinct datasets were created by varying these parameters, each representing a unique data processing method. 

To understand the impact of parameter adjustments on the statistical outcomes, principal component analysis (PCA) and orthogonal partial least squares discriminant analysis (OPLS-DA) were applied to compare the quality of the resulting models. The goodness of fit of the models was assessed using metrics such as R^2^X, which indicates the proportion of variance in the data that is explained by the model, and Q^2^, which measures the predictive capability of the model. These metrics provide insight into how well the data matrices align with the underlying structure of the dataset, and their improvement upon parameter optimization indicates the effectiveness of the preprocessing steps [11]. The obtained results are presented in Table 1. Our results indicate that the highest R^2^X and Q^2^ values in PCA models were observed for datasets obtained in positive ionization rather than negative, applying highest intensity threshold investigated (5000) rather than lower, using QC samples, batch correction rather than no correction. Impact of different chromatographic separation was less clear and varying with ionization mode; a shorter gradient (30 min) was more beneficial in ESI−, longer (37 min) in ESI+.

In our study, we compared datasets processed using positive ionization (ESI+) and negative ionization (ESI−). The analysis revealed that PCA models generated with ESI+ consistently exhibited higher R^2^X and Q^2^ values compared to those generated with ESI−, indicating that positive ionization provided better model quality and predictive power. Based on the research conducted so far, it is known that sometimes negative ionization is a better option due to its improved sensitivity [20]. On the other hand, the study by Okińczyc et al. indicates that some compounds, such as pinostrobin or tectochrysin, ionize weakly in ESI−, and therefore will not be detected [21]. This may suggest that in positive ion mode the noise level increases due to the greater number of compounds that ionize.

The parameter that directly affects the noise level is the intensity threshold. Intensity thresholds determine the minimum signal intensity required for a peak to be considered valid for analysis. Selection of them is a delicate task, as it is important to balance the exclusion of noise while maintaining the integrity of significant signals [22]. According to research of Tugizimana et al. [11], where two intensity thresholds—10 and 100—were tested, higher threshold not only reduces the noise level, but also gives higher R^2^X and Q^2^ values in the PCA model, thus improving its quality. In this study, we tested four different intensity thresholds: 500, 1000, 2000, and 5000. The analysis showed that the highest R^2^X values were obtained for models with an intensity threshold of 5000, suggesting that higher thresholds are beneficial for reducing noise and improving model quality. However, the highest Q^2^ values were observed for models with an intensity threshold of 2000, indicating that a moderate threshold may provide better predictive accuracy. In a study conducted by Houriet et al. [22], in which nine different intensity thresholds from 1 × 10^2^ to 1 × 10^6^ were tested, it was concluded that the optimal threshold values should be adapted to the analysis objective—for simple comparative analyses high intensity thresholds are sufficient, however, for more detailed compound identification lower thresholds are preferable, as they increase the number of detected features, improving the limit of detection (LOD) and compound identification (isotope patterns, MS/MS spectra). 

In our study, we also investigated how different chromatographic separations (different gradients) affect the quality of metabolomics studies. Changing the gradient affects the separation of compounds and the duration of the analysis. While shorter analyses provide substantial reductions in time and use of consumables, poorer chromatographic separation may lead to interference between analytes and distortion or loss of some information. This may happen by affecting compound ionization (ion suppression or enhancement) or interference in automatic MS feature detection processes due to loss of chromatographic resolution. Based on our results, a longer gradient (37 min) providing slightly better separation was found to be advantageous for ESI+ datasets, producing higher R^2^X and Q^2^ values in most cases. This suggests that altering the gradient and extending the separation time enabled better fractionation and resolution of metabolites, leading to more accurate models. On the other hand, for ESI− datasets, data obtained by shorter gradient separation (30 min) yielded superior results. 

QC (quality control) refers to the use of control samples in analyses, aiming at monitoring the stability and precision of the LC-MS system. Pooled QC samples, which are a mixture of all samples analyzed in a given experiment, are often used to assess the quality of the analysis and identify possible systematic errors [23]. Our results showed that models based on buckets obtained using QC samples batch correction consistently had higher R^2^X and Q^2^ values compared to those without QC samples. This reinforces the idea that QC samples are essential for identifying and correcting systematic errors in the data, thus enhancing the reliability and robustness of analyses [23].

In addition to investigating the effect of changing preprocessing parameters on the quality of statistical models, this study also evaluated the influence of scaling. Different scaling methods were tested, including centering, no scaling, unit variance, unit variance normalization, Pareto, and Pareto normalization. The obtained results are presented in Table 2. The highest R^2^X and Q^2^ values in PCA models were obtained without scaling for both positive and negative ionization modes and different retention times. This suggests that not applying any scaling provided a more accurate representation of the data, leading to higher quality models. These results are consistent with those obtained by Tugizimana et al., where among the compared scaling methods, the highest R^2^X and Q^2^ values were also achieved by models without scaling [11]. Based on these results, a consistent conclusion can be drawn that scaling significantly affects the model’s goodness of fit and its predictive ability.

### 2.2. Principal Component Analysis (PCA)

**Figure 1:**
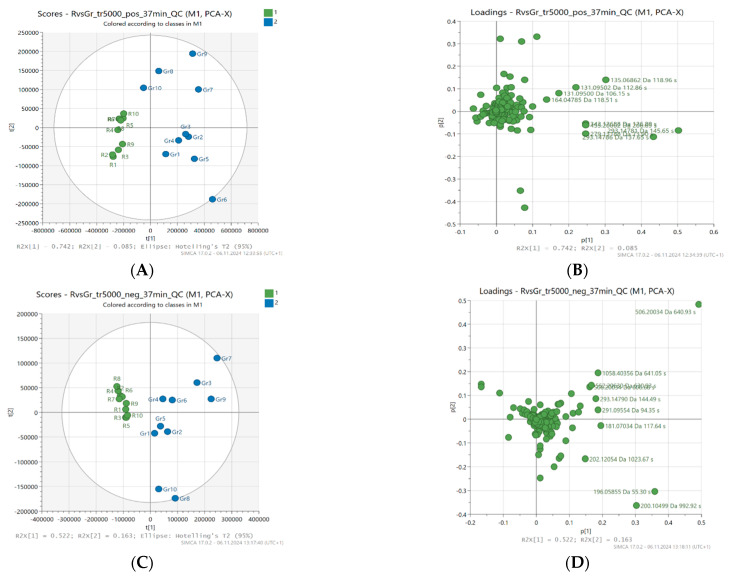
Principal component analysis (PCA) scores (**A**,**C**) and loading plots (**B**,**D**), not scaled; based on LC-MS fingerprints recorded in ESI+ (top) and ESI− (bottom) for 20 honey samples (buckwheat—blue, canola—green) characterized by different levels of antioxidant activity.

Principal component analysis (PCA) was used to assess the distribution of samples and the contribution of variables to the creation of principal components. PCA scores (A and C) and PCA loadings (B and D) of the best results, a 37-min gradient, and a threshold of 5000 were used, and are presented in Figure 1.

As described in the experimental section, PCA was performed separately for data sets obtained from LC-MS with positive and negative ionization. Twenty samples and 555 or 343 variables, respectively, were used and reduced to two principal components (PC). In positive ionization, these components accounted for 82.7% of the total variance in the analyzed honey samples (PC1 accounted for 74.2% of the variance, and PC2 8.5%). In turn, for negative ionization, the first two factors explained 52.2% (PC1) and 16.3% (PC2), respectively, which together constituted 68.5% of the total variance between samples.

In the PCA scores analysis, attention was paid to the spatial arrangement of samples that were characterized by a similar level of antioxidant activity and botanical origin. The group of samples showing high antioxidant activity was located on the right side of the graph and in almost all cases showed positive values for PC1 in both ESI+ and ESI−. It was clearly separated from samples with low antioxidant activity (e.g., FRAP ≤ 1.6 mmol Fe^2+^/kg), which in turn were grouped on the left side of the graph. The corresponding PCA loadings plots (Figure 1) present the contribution of individual variables (metabolites) to the creation of the principal components.

### 2.3. Orthogonal Projections to Latent Structures (OPLS-DA)

To assess the ability to discriminate between honey samples with different antioxidant activity and to identify potential metabolites contributing to antioxidant activity according to the correlation between metabolites (x variables) and antioxidant activity (y variables), orthogonal partial least squares discriminant analysis (OPLS-DA) was performed. Ten samples with higher antioxidant activity (FRAP > 1.6 mmol Fe^2+^/kg) and ten samples with lower activity (FRAP ≤ 1.6 mmol Fe^2+^/kg) were used for the analysis, and the data were centered accordingly before modeling.

**Figure 2:**
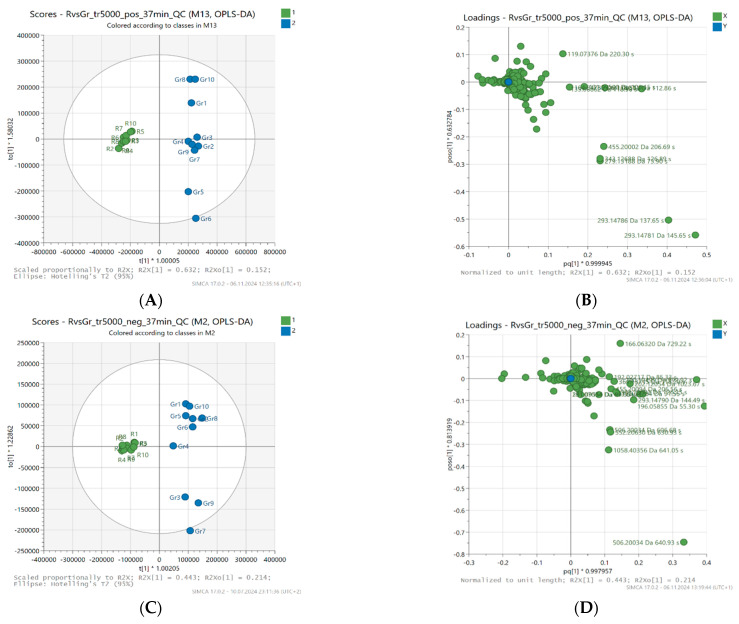
Orthogonal projections to latent structures (OPLS-DA) scores (**A**,**C**) and loading plots (**B**,**D**), centered, based on LC-MS fingerprints recorded in ESI+ (top) and ESI− (bottom) for 20 honey samples (buckwheat, canola) characterized by different levels of antioxidant activity.

Figure 2 shows the OPLS-DA scores and loading plots, enabling a better understanding of the structure and variability of the data. In the score plot (A and C) for the predictive component (t [1]), a clear separation is noticeable between two groups of samples—active (blue) and less active (green). The t [1] component accounts for 63.2% of the variability in the positive ionization data (R^2^X [1] = 0.632) and 44.3% of the variability in the negative ionization data (R^2^X [1] = 0.443), indicating that the model effectively separates both groups based on this principal component.

In addition, the model achieved high R^2^Y values (0.989 for positive ionization data and 0.957 for negative ionization data), indicating a good fit of the model to the data and reliability. The Q^2^ value (0.928 in positive and 0.914 in negative ionization) also confirms the high predictive ability of the model, indicating its reliability. As a result, the OPLS-DA classification model shows high accuracy, with a success rate of 100% in both cases, confirming that it was able to effectively distinguish between honey samples with higher and lower antioxidant activity.

The reliability of the computed OPLS-DA models was assessed by performing ANOVA-CV and validated by permutation tests to minimize the risk of over-fitting. For all 20 data sets, the *p*-values of the computed OPLS-DA models were significantly lower than 0.05, which indicates the high statistical quality of the models. On the other hand, the permutation test results show that the R^2^ and Q^2^ values for the true models were much higher than those for the 100 permuted models, which proves that the computed OPLS-DA models are statistically significant for all data sets and the obtained results are not random.

**Figure 3:**
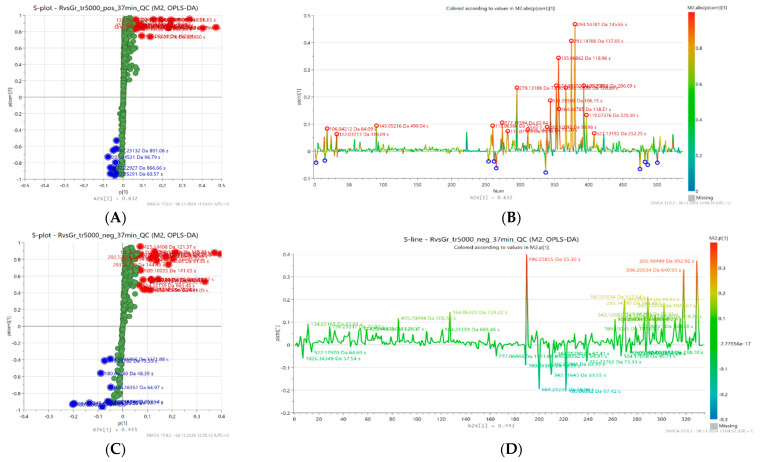
Orthogonal partial least squares discriminant analysis (OPLS-DA) S-plots (**A**,**C**) and S-lines (**B**,**D**) based on LC-MS fingerprints recorded in ESI+ (top) and ESI− (bottom) for 20 honey samples characterized by different levels of antioxidant activity. Selected features related to low (blue) or high (red) antioxidant activity are highlighted.

To explore the relationship between metabolites and antioxidant activity levels, visualization of variables (retention time and exact mass pairs) was used by creating an S-line and an S-plot, shown in the Figure 3. The S-line plot (B and D) enabled the evaluation of the effect of individual variables on sample separation in the model space, while the S-plot (A and C) analysis allowed for the identification of metabolites that were particularly associated with high antioxidant activity. In the S-plot, variables (metabolites) correlating with high antioxidant activity (characterized by high magnitude (modeled co-variation) and high reliability (modeled correlation)) are highlighted in the upper right corner (red color), while those associated with low activity are in the lower left corner (blue color).

In the model based on the positive ion mode (ESI+), 20 features associated with increased antioxidant activity were distinguished, while in the model based on the negative ion mode (ESI−) 23 features were selected. Four metabolites were common to both models. The extracted compounds can be considered as potential markers of high antioxidant activity and their details are presented in the Table 3. The most relevant group was represented by Amadori compounds, particularly N-fructosyl derivatives of amino acids—tyrosine, valine, leucine, isoleucine, phenylalanine, pyroglutamic acid, and proline. Major groups were also amino acids related to the previous group (except phenylalanine) as well as organic acids (gluconic, malic, citric, phenyllactic and sebacic acid). Other compounds included various nitrogen-containing heterocycles (glucopyranosylfagomine, indoline, 4-hydroxyquinoline), linden glycoside isomers, and aldehydes (salicylaldehyde, benzaldehyde).

### 2.4. Identification of Marker Compounds Associated with Increased Antioxidant Activity

**Figure 4:**
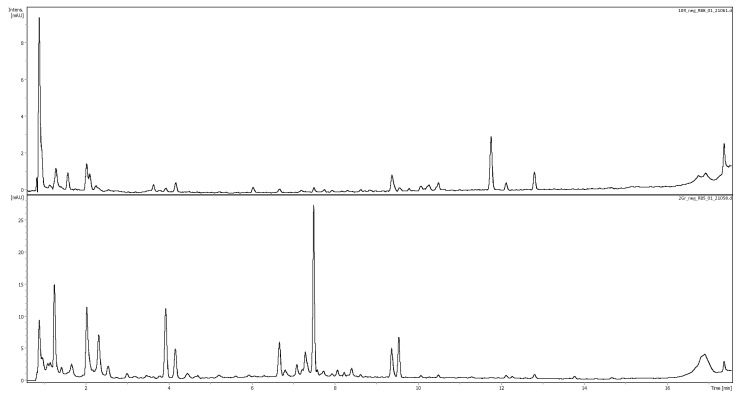
Comparison of representative chromatographic profiles of canola (**top**) and buckwehat (**bottom**) honeys recorded at 280 nm.

The potential marker compounds were identified (with varying levels of confidence) based on comparison with analytical standard and retention time, exact mass, MS^2^ fragmentation, and UV spectra, the literature data, and/or MS^2^ databases (Bruker MetaboBASE Personal Library 3.0, MS Dial library (MSMS-Public-VS15) and LC-MS/MS MoNA library). The analysis enabled the identification or putative identification of 18 of the 23 selected mass-retention time pairs corresponding to 16 compounds in the negative ionization, as well as 18 of the 20 mass-retention time pairs corresponding to 18 metabolites in the positive ionization. The identification results are presented in Table 3. Figure 4, below, shows a chromatogram of buckwheat honey recorded at 280 nm with a canola honey profile for comparison.

In negative ionization, a large part of the identified compounds are organic acids such as malic acid, phenyllactic acid (PLA), gluconic acid, citric acid, pyroglutamic acid, and sebacic acid. The presence of the first four of these in buckwheat honey was confirmed in this study by comparison with analytical standards. Based on the studies conducted so far, it is known that gluconic acid is the dominant non-aromatic acid found in honeys [24]. Malic acid, citric, pyroglutamic and sebacic acids are also found in abundance [24,25]. Gluconic, malic, and citric acids are known for their ability to chelate metals, thus contributing to the antioxidant activity of honey [26]. PLA is commonly found in thistle (*Galactites tomentosa* Moench) unifloral honeys, heather, ling heather, and manuka honeys [27]. However, it has not been indicated as characteristic of buckwheat honey so far.

In positive ionization, the most important group of metabolites associated with increased antioxidant activity of buckwheat honey in this study turned out to be Amadori products, intermediate products of the Maillard reaction, formed in the condensation reaction between the carbonyl group of a reducing sugar (in this case fructose) and the –NH_2_ group of an amino acid. These compounds are subsequently transformed into melanoidins [7].which have antioxidant activity resulting from radical-scavenging capacity [8]. Several Amadori compounds were detected and identified, in both positive and negative mode: N-fructosyl-Tyr, N-fructosyl-Ile and N-fructosyl-Ile/Leu hex, while N-fructosyl pyroglutamate (derivative of a non-proteinogenic amino acid) in ESI− and i.a. N-fructosyl-Pro, N-fructosyl-Val in ESI+ were additionally observed. Interestingly, to the best of our knowledge, this is one of the first reports on these Amadori compounds in buckwheat honey. The MS^2^ fragmentation of these compounds with one monosaccharide follow similar pattern with base peak corresponding to the 162 amu loss corresponding to hexose and other fragments corresponding to fragmentation of amino acids [28]. For example base peaks in MS^2^ of N-fructosyl pyroglutamate (*m/z* 290.0890), N-fructosyl tyrosine (*m/z* 342.1209), N-fructosyl isoleucine (*m/z* 292.1410) correspond to appropriate amino acids after loss of sugar moiety [M-H-C_6_H_10_O_5_]: (*m/z* 128.0337; 180.0665; 130.0869 corresponding to pyroglutamic acid, tyrosine, isoleucine).

These results are supported by the fact that sugars represent a major part of every honey. Similarly, amino acids are also compounds commonly found in honey, but with more variable proportions [29]. In this study, we detected amino acids not only bound to fructose as Amadori compounds, but also in free form. Three metabolomic features can be assigned to tyrosine: 181.07034 Da (RT = 1.96 min) and 361.13715 Da (RT = 1.97 min) corresponding respectively to *m/z* 180.0669 and *m/z* 360.1325 in negative ionization, as well as 135.06862 Da (RT = 1.98 min) corresponding to *m/z* 182.08132 in positive ionization. The assignment of m/z 181 to mass 135 Da results from an incorrect interpretation by the program, which identified this mass as a formic acid adduct. The *m/z* 360 corresponds to twice the mass of the tyrosine pseudomolecular ion in negative ionization (*m/z* 180) and can be interpreted as an artifact created during MS analysis. This hypothesis is supported by the fact that this mass in MS^2^ decays to a mass corresponding to the mass of tyrosine. Moreover, retention time, MS and UV spectra corresponded to those of pure tyrosine standard. This highlights that proper assignment of compounds to metabolomics features requires careful attention. In positive ionization, amino acids such as L-proline, valine, L-isoleucine and L-leucine were also identified. These results are consistent with outcomes of Dimins et al., indicating tyrosine, isoleucine, and leucine as potential markers of buckwheat honey [30], as well as recent research of Yan Zhu et al., highlighting exceptionally high content of tyrosine and branched-chain amino acids (BCAA)—valine, leucine, isoleucine in buckwheat honey in contrast to other honeys [31].

Compounds characterized by masses of 106.04212 Da (RT = 84.09 s) and 122.03713 Da (RT = 118.09 s) belong to aldehydes and are identified as benzaldehyde and salicylaldehyde (detected in positive ionization). The first of them has been already identified in buckwheat honey [32], while the second one is considered a probable marker of pine honey. However, it is also known that salicylaldehyde is responsible for the characteristic aroma of buckwheat, therefore, its presence in buckwheat honey is possible [33].

In positive ionization, an alkaloid 4-hydroxyquinoline (145.0522 Da 8.17 min) was detected and confirmed with standard compound. It has been previously identified in Castanopsis honey, *Dendropanax dentiger* honey, chestnut honey and jujube honey [34,35]. It exists in equilibrium with its major tautomer, 4-quinolone, found also in buckwheat honey [36]. Other detected compound was 3′,4′-(methylenedioxy)acetophenone. Acetophenone and its derivatives have already been detected in honeys [37], therefore the presence of 3′,4′-(methylenedioxy) acetophenone (164.04785 Da 1.98 min) in buckwheat honey is possible. 

A group of potential markers characterized by RT 10.11–10.68 min and masses 506.20034, 506.20034, 552.20630 and 1058.40356 were identified as three linden glycosides. The two later masses correspond to [M + FA] and [2M + FA] adducts incorrectly recognized as compound mass. Their identity was confirmed by comparison with other honey types, these compounds were identified previously in linden honey and also some other varieties [38].

Compound characterized by mass 180.06351 was tentatively identified as D-tagatose based on exact mass and UV maximum (284 nm) corresponding to literature data [39] considering that this monosaccharide was previously found in buckwheat nectar [40]. Compound at RT 1.32 min and mass 309.14243 was tentatively identified as glucopyranosyl fagomine, considering the presence of *m/z* 148 corresponding to fagomine in MS^2^ spectrum. Fagomine has previously been identified in buckwheat seeds [41].

### 2.5. Antioxidant Activity and Total Phenolic Content

The antioxidant activity of honeys was measured using the DPPH test for free radical scavenging activity, and the FRAP test, which assesses the ability to reduce Fe^3+^ to Fe^2+^. Total phenolic content (TPC) was also measured. The latter is sometimes also used to assess antioxidant potential as it is based on redox reaction [42,43]. Therefore, a cautious and critical approach to this method as assessment of phenolic content it is important. The results, presented as mean ± standard deviation, are presented in the Table 4. All tests performed confirmed significantly higher antioxidant activity of buckwheat honey compared to canola honey and the phenolic content in buckwheat honey was also higher than in canola honey. Student’s *t*-test showed that these differences are statistically significant (*p* < 0.001 for all three tests). The total phenolic content for buckwheat honey reached nearly 1000 mg GAE/kg, while that for canola was slightly above 100 mg GAE/kg. The disproportion in reducing power and antiradical activity was slightly lower and was about fivefold in favor of buckwheat honey (about 6 vs. 1 mmol Fe^2+^/kg—FRAP; about 61 vs. 12 mg GAE/kg -DPPH). These results are consistent with the results of previous studies that showed much greater antioxidant activity and total phenolic content of buckwheat honey than canola honey based on DPPH [1,16,44], FRAP [44], and TPC [1,16,44] assay.

The Pearson correlation analysis showed positive, statistically significant correlations between total phenolic content and radical scavenging activity (r = 0.967), total phenolic content and ferric reducing antioxidant power (r = 0.668) and between radical scavenging activity and ferric reducing power (r = 0.540). This observation is consistent with previous research focusing on a number of different honey types [45].

What is more, the Pearson correlation analysis revealed numerous statistically significant (*p* < 0.05), positive relationships between the content (ion abundance) of selected metabolites and total phenolic compounds, antioxidant capacity, measured using Folin Ciocalteu (TPC), DPPH, and FRAP assays. The results are presented in Table 5 and examples of correlations in Figure S1. Of the identified compounds, only malic acid did not show any statistically significant correlation between its content in honey and antioxidant activity. Several compounds showed consistently high correlation coefficients in all antioxidant tests, suggesting their importance as potential markers of increased antioxidant activity. High correlation of TPC with compounds that do not have phenolic group draws attention and reminds that careful use of Folin–Ciocalteu assay, as a method to determine phenolic content, is required since this reagent may also react with other compounds. It is therefore necessary to look critically at the results attributing the antioxidant properties of honeys to phenolic compounds on the basis of correlation with this test [3]. Some of the highly correlated compounds are known as non-phenolic antioxidants, which may support usage of this test as antioxidant assay in honey research rather than estimation of phenolic content.

The metabolites showing strong correlations included organic acids such as pyroglutamic acid and citric acid. This is consistent with studies conducted so far, which indicated high antioxidant activity of citric acid [30]. Aldehydes generally have not been associated with antioxidant activity so far, but in our study benzaldehyde showed a very strong correlation with reducing power (FRAP), radical scavenging activity (DPPH), and total phenolics content, slightly less so for salicylaldehyde. Significant correlation of some other compounds does not necessarily mean that they are meaningful from the point of view of antioxidant activity. Positive correlation may be also related to chemical characteristics of the active variety and thus play the role of an analytical marker rather than active marker. Therefore, further research is needed to verify the relevance of individual compounds and their potential synergies. The very strong, positive correlation of tyrosine (R > 0.96) with the results of all tests is noteworthy. Folin–Ciocalteu assay originally was applied to quantify this amino acid [46]. In addition, among free amino acids, very strong correlations were also observed for leucine, isoleucine, proline and valine. So far, research has mainly pointed to the antioxidant activity of sulfur-containing amino acids which are involved in the synthesis of intracellular antioxidants such as glutathione [47,48]. The correlation itself does not necessarily indicate the activity of individual compounds, but may be due to correlation with its active derivatives.

The strong correlations were also observed for Amadori compounds such as N-fructosyl-isoleucine, N-fructosyl-leucine, N-fructosyl-tyrosine, N-fructosyl-valine, N-fructosyl-proline, N-fructosyl-phenylalanine and are particularly noteworthy. These early Maillard reaction products are formed by non-enzymatic condensation of reducing sugars with amino groups of amino acids [7]. In addition to their importance as processing indicators, several Amadori products have been shown to have antioxidant properties. For example, in thermally processed foods such as black garlic, these compounds have been directly linked to increased antioxidant potential [49]. Their antioxidant activity is known to have an inhibitory effect on the oxidation caused by metal ions because they are effective transition metal ions chelators, possess Fe^3+^ ion reducing ability, hydroxyl radical scavenging ability and may inhibit oxidative degradation of DNA caused by Cu^2+^ ion [50]. Considering the compounds identified in buckwheat honey, according to Zhou et al., Fru-Val exhibits moderate Fe^2+^ chelating ability (above 40%), Fru-Phe exhibits moderate Fe^2+^ and Cu^2+^ chelating ability (near 40%) at 10 mmol/L, while Fru-Pro had one of the best Fe^3+^ reducing ability (0.30 mmol/L of ascorbic acid equivalent). Moreover, the hydroxyl radical scavenging ability for Fru-Leu, Fru-Pro, Fru-Val, and Fru-Phe ranged from about 20 to 35% at 10 mmol/L and the inhibition rate of DNA oxidative damage in Fenton-like reaction by Fru-Phe reached 43.11% at 10 mmol/L [50]. These activities are considerable higher than those of the corresponding amino acids, which may suggest that significant correlations of amino acids with antioxidant activity may be rather related to the fact that they are Amadori products substrates. 

Amadori products are also known precursors of melanoidins, high molecular weight nitrogen polymers formed in the later stages of the Maillard reaction [49] Melanoidins have been identified as the major radical scavenging components in heat-treated foods such as honey, contributing significantly to their antioxidant activity [7,8]. While melanoidins themselves are not readily detectable by typical metabolomic approaches due to their polymeric nature, the presence of Amadori intermediates in the analyzed samples may indirectly reflect the extent of Maillard reaction progression and melanoidin formation. Moreover, the fact that these low molecular weight Amadori compounds correlate so strongly with antioxidant activity supports the hypothesis that they may exert their own biological effects, independent of melanoidins. The latter escape gastrointestinal digestion and are fermented by the gut microbiota which affects their antioxidant capacity [51]. The relatively small size of these Amadori products suggests that they may also have improved bioavailability, which would further enhance their nutritional and functional importance in the human diet. This is supported by research of de la Cueva et al. demonstrating higher transport rates of lower molecular weight fractions through Caco−2 cell monolayers [52]. The absorption through diffusion is proposed as a mechanism and bioavailability level varies from weak to very good between specific compounds [53,54,55,56]. Regardless of intestinal absorption rate, they could also contribute to local improvement of oxidative stress measures when eaten or wherever it is applied—in the intestine, throat or wounded skin. Therefore, both Amadori products and their potential transformation into melanoidins may be important not only from a chemical perspective but also in terms of bioactivity and health-related outcomes. Further research is needed to assess potential biological activity of individual compounds in vivo. Amadori products in honey may also be relevant from the point of view of food science and food authentication. They contribute to taste (such as sweet, bitter, sour, salty, umami, kokumi) and flavor of food [57]. Their identification in honey may contribute to better understanding of sensory characteristics of its different varieties. Therefore, differences in Amadori products composition in various honey types may also be particularly attractive for honey authentication.

## 3. Materials and Methods

### 3.1. Chemicals and Instruments

Acetonitrile (both gradient grade and LC-MS grade), LC-MS grade water, along with formic acid were sourced from Merck (Sigma-Aldrich, Steinheim, Germany). Ultrapure water (<0.06 μS/cm) was obtained from the Hydrolab HLP20UV purification system (Hydrolab, Straszyn, Poland). The standards for gallic acid, ferrous sulfate, 1,1-diphenyl-2-picrylhydrazyl radical (DPPH), 2,4,6-tris(2-pyridyl)-1,3,5-triazine (TPTZ), and ferric chloride were also purchased from Merck (Sigma-Aldrich, Steinheim, Germany). Folin-Ciocalteu reagent and Na_2_CO_3_ were acquired from Avantor Performance Materials Poland (POCH, Gliwice, Poland).

### 3.2. Sample Preparation

Honey samples used for the study came from Polish beekeepers who declared their floral origin. It was further confirmed by assessment of their characteristic chromatographic fingerprints. 10 samples of buckwheat (*Fagopyrum esculentum* Moench) honey and 10 samples of canola (*Brassica napus* L. var. oleifera Metzger) honey were analyzed. They were stored at 4 °C with no exposure to light, prior to analysis, diluted with ultrapure water (1:5, *w/v*) using ultrasound treatment and then filtered through PHENEXTM 0.2 µm, Ø25 mm, PTFE syringe filters (Phenomenex, Torrence, CA, USA). A QC sample was created by mixing equal volumes of each sample.

### 3.3. LC-MS Analysis

The raw dataset was acquired through LC-MS analysis of honey samples. Chromatographic separation was performed using a Thermo Scientific UltiMate 3000 UHPLC system (Thermo Scientific™ Dionex™, Sunnyvale, CA, USA), equipped with an autosampler, a temperature-controlled column compartment, DAD detectors, and a QqTOF-MS system. A sample volume of 5 µL was injected onto a Kinetex^®^ C18 2.6 µm, 100 Å, 150 × 2.1 mm analytical column with a guard column (Phenomenex, Torrence, CA, USA), maintained at a temperature of 35 ± 1 °C. The mobile phase for chromatographic separation consisted of aqueous 0.1% formic acid (solvent A) and acetonitrile (solvent B). Its flow rate was set at 0.4 mL/min. The gradient elution program commenced with 100% solvent A, which was gradually reduced to 91% over 7 min and maintained for an additional 3 min. After 10.5 min, the percentage of solvent A was further decreased to 80%, and after another 8 min, it was reduced to 60%. The mixture was held isocratic until 22.5 min, followed by the solvent A content decrease to 0% at 28.5 min, and maintained isocratic until 32 min. Finally, the conditions were returned to the initial state and stabilized prior to the next injection. 

Several QC injections were analyzed at the beginning, at the end, and every 10 samples. DAD detector settings included acquisition of full spectra in the range of 200–600 nm as well as selected wavelengths: 220, 254, 280, 320, and 360 nm. MS and MS^2^ analyses were performed using a compact QqTOF-MS detector (Bruker, Darmstadt, Germany), which was used in negative and positive ESI modes. The ion source temperature was set at 100 °C, the nebulizer gas pressure at 2.0 bar, the dry gas flow at 0.8 L/min, and the temperature at 210 °C. The capillary voltage were 2.20 kV (negative mode) or 4.50 kV (positive mode), and the collision energy 8.0 eV. The ESI−MS^2^ experiments were performed at 35 eV collision energy and N_2_ as collision gas. Internal calibration was achieved by injecting a 10 mM solution of sodium formate clusters.

### 3.4. Chemometric Analysis of the LC-MS Fingerprints

For data preprocessing, the MetaboScape^®^ 2021b software (Bruker, Billerica, MA, USA) was used. This step includes noise filtering, automatic peak detection, baseline correction, data binning, deconvolution and chromatographic alignment [12,18]. By changing the gradient, ionization, inclusion of QC sample injection in LC-MS and intensity threshold settings in the data processing step, 20 data matrices were obtained. They were created taking into account the QC sample injection and, for comparison, 4 matrices were created without the QC. Four intensity thresholds were tested in the data cleaning step: 500, 1000, 2000 and 5000 for analyses with QC, and for those without QC sample injection—an intensity threshold of 1000.

Created matrices were exported to Simca^®^ v. 17.02.34594 (Sartorius Stedim Data Analytics AB, Malmö, Sweden) for statistical analysis. In the first step, principal component analysis (PCA) was used to investigate the overall structure of the data and compare different data preprocessing techniques. Different scaling methods were compared and centering was chosen as the scaling method to compare impact of intensity threshold and other parameters on model performance. To further investigate the differences between samples with different levels of antioxidant activity and to determine the associated chemical markers, orthogonal projections onto latent structures analysis (OPLS-DA) was performed. In order to visualize the significance of each variable in the model, an S-line plot was created, as well as an S-plot, which revealed correlations between variables, indicating potential biomarkers (retention time and exact mass pairs) associated with high or low antioxidant activity. Compound identification was based on retention time, exact mass, MS fragmentation, and comparison with analytical standards. The HMDB and MetaboScape MS^2^ databases were used.

### 3.5. Statistical Analysis

Statistical analyses were performed using the software Statistica 13 (TIBCO software Inc., Palo Alto, CA, USA). Pearson’s correlation between the investigated parameters and significance was assessed in two-tailed test at the level of significance *p* < 0.05. Student’s *t*-test was used to verify significant differences between the means of two groups.

### 3.6. Antioxidant Activity Tests

A BioTek Epoch 2 Microplate Spectrophotometer (Agilent, Santa Clara, CA, USA) was applied for total phenolics, DPPH, and FRAP determinations. The spectrophotometric measurements were performed in triplicate using a polystyrene 96-well plates (Sarstedt, Nümbrecht, Germany). 

#### 3.6.1. Total Phenolic Content (TPC)

TPC in honey was determined by the modified Folin–Ciocalteu method [58]. 200 μL of the aqueous sample solution was mixed with 40 μL of Folin–Ciocalteu reagent and allowed to react for 5 min. Then 800 μL of 100 g/L (*w/v*) Na_2_CO_3_ was added. The absorbance was measured at 725 nm against a blank sample after 50 min of incubation at room temperature. Total phenols content, expressed in milligrams of gallic acid equivalent (GAE) per kilogram, was calculated from a calibration curve prepared from a standard gallic acid solution (2.5–100 μg/mL), which was analyzed in the same way as the honey samples.

#### 3.6.2. Antiradical Activity (DPPH Assay)

The antiradical activity of the analyzed honeys was measured using DPPH assay, as described in the literature [59]. For this purpose, 50 µL of the aqueous honey solution (1:5, *w/v*) was mixed with 200 µL of 0.03 mmol/L DPPH in methanol in the wells of a 96-well microtiter plate and left at room temperature with no exposure to light for 30 min. After the incubation, the absorbance measurements were performed at 517 nm against a blank. Results were calculated based on a calibration curve prepared with gallic acid solution (2.5–100 μg/mL) and expressed as gallic acid equivalent antioxidant capacity per kilogram of the honey (mgGAE/kg).

#### 3.6.3. Total Antioxidant Activity (FRAP Assay)

For the analysis of ferric reducing antioxidants, a modified FRAP method was employed [60]. The reagent was made by combining 10 mmol/l TPTZ with 20 mmol/L iron chloride in acetate buffer (pH 3.6). Then, 20 µL of aqueous honey solution (1:5, *w/v*) was mixed with 200 µL of the prepared FRAP reagent. After incubating for 4 min, the absorbance was measured at 593 nm against a blank sample. The ferric reducing antioxidant power, expressed as millimoles of Fe^2+^ per kilogram of honey, was calculated using the external standard method. The calibration curve was prepared with iron sulfate solutions (0.1–2 mmol/L).

## 4. Conclusions

This study highlights the potential of LC-MS-based untargeted metabolomics combined with antioxidant assays to explore the chemical basis of buckwheat honey’s bioactivity. One of the key outcomes was the optimization of data preprocessing, which demonstrated that the use of positive ionization mode, the inclusion of QC samples, and the absence of scaling yielded the most robust statistical models (PCA and OPLS-DA), with the highest R^2^X and Q^2^ values. When it comes to the chromatographic gradient, the longer method (37 min) gave better model quality when using ESI+, and the shorter one (30 min) when using ESI−. Additionally, intensity thresholds of 2000–5000 counts were shown to balance noise reduction with signal retention, further improving model reliability.

OPLS-DA modeling enabled the identification of 43 metabolites strongly associated with increased antioxidant activity. Among them, several organic acids (citric, pyroglutamic), aldehydes (benzaldehyde), free amino acids (tyrosine, isoleucine, leucine), and Amadori compounds (e.g., N-fructosyl-tyrosine, N-fructosyl-isoleucine) were recognized as potential chemical markers of antioxidant potency. Notably, the presence of Amadori products supports the hypothesis that Maillard reaction intermediates and melanoidin precursors significantly contribute to the antioxidant profile of buckwheat honey. These findings not only expand the current understanding of honey bioactivity but also offer a validated methodological approach for future studies aiming to identify functional markers in complex natural products. Nevertheless, due to limitations of current study, further research including larger sample size, validation of the identified compounds, their activity and metabolic pathways in vivo is needed to verify and better understand the role of particular components to antioxidant activity of buckwheat honey. 

## Figures and Tables

**Table 1:** PCA and OPLS-DA models created using centering as the scaling method and different ion mode (positive/negative), intensity threshold (500/1000/2000/5000 counts), chromatographic separation (30 min/37 min gradient), QC batch correction inclusion/no inclusion.

Model	QC ^2^	Ion Mode ^3^	Gradient (min)	Treshold (Counts)	Type	R^2^X (cum)	R^2^Y (cum)	Q^2^ (cum)	Permutation (100)	C-A ^1^ (*p*)
*The influence of QC inclusion, negative ionization and 30 min gradient on statistical models*										
1.	+	−	30	500	PCA-X	0.672		0.370		
					OPLS-DA	0.656	0.962	0.919	R^2^ = (0.0. 0.272) Q^2^ = (0.0. −0.509)	5.34 × 10^−8^
2.	+	−	30	1000	PCA-X	0.753		0.682		
					OPLS-DA	0.659	0.961	0.917	R^2^ = (0.0. 0.296) Q^2^ = (0.0. −0.438)	6.37 × 10^−8^
3.	+	−	30	2000	PCA-X	0.758		0.690		
					OPLS-DA	0.661	0.961	0.917	R^2^ = (0.0. 0.286) Q^2^ = (0.0. −0.392)	6.33 × 10^−8^
4.	+	−	30	5000	PCA-X	0.765		0.689		
					OPLS-DA	0.662	0.962	0.920	R^2^ = (0.0. 0.288) Q^2^ = (0.0. −0.418)	4.75 × 10^−8^
*The influence of QC inclusion, negative ionization and 37 min gradient on statistical models*										
5.	+	−	37	500	PCA-X	0.737		0.668		
					OPLS-DA	0.625	0.958	0.902	R^2^ = (0.0. 0.373) Q^2^ = (0.0. −0.449)	2.12 × 10^−7^
6.	+	−	37	1000	PCA-X	0.745		0.678		
					OPLS-DA	0.627	0.957	0.902	R^2^ = (0.0. 0.373) Q^2^ = (0.0. −0.383)	2.07 × 10^−7^
7.	+	−	37	2000	PCA-X	0.751		0.683		
					OPLS-DA	0.641	0.955	0.906	R^2^ = (0.0. 0.324) Q^2^ = (0.0. −0.545)	1.54 × 10^−7^
8.	+	−	37	5000	PCA-X	0.685		0.400		
					OPLS-DA	0.657	0.957	0.914	R^2^ = (0.0. 0.316) Q^2^ = (0.0. −0.488)	7.84 × 10^−8^
*The influence of QC inclusion, positive ionization and 30 min gradient on statistical models*										
9.	+	+	30	500	PCA-X	0.748		0.676		
					OPLS-DA	0.853	0.983	0.898	R^2^ = (0.0. 0.477) Q^2^ = (0.0. −0.769)	9.39 × 10^−6^
10.	+	+	30	1000	PCA-X	0.756		0.684		
					OPLS-DA	0.854	0.983	0.898	R^2^ = (0.0. 0.502) Q^2^ = (0.0. −0.616)	9.48 × 10^−6^
11.	+	+	30	2000	PCA-X	0.810		0.645		
					OPLS-DA	0.854	0.983	0.898	R^2^ = (0.0. 0.494) Q^2^ = (0.0. −0.681)	9.48 × 10^−6^
12.	+	+	30	5000	PCA-X	0.815		0.650		
					OPLS-DA	0.859	0.983	0.899	R^2^ = (0.0. 0.465) Q^2^ = (0.0. −0.632)	8.83 × 10^−6^
*The influence of QC inclusion, positive ionization and 37 min gradient on statistical models*										
13.	+	+	37	500	PCA-X	0.790		0.732		
					OPLS-DA	0.894	0.990	0.927	R^2^ = (0.0. 0.714) Q^2^ = (0.0. −0.688)	6.40 × 10^−6^
14.	+	+	37	1000	PCA-X	0.804		0.748		
					OPLS-DA	0.897	0.989	0.925	R^2^ = (0.0. 0.714) Q^2^ = (0.0. −0.624)	7.24 × 10^−6^
15.	+	+	37	2000	PCA-X	0.810		0.756		
					OPLS-DA	0.898	0.989	0.926	R^2^ = (0.0. 0.7) Q^2^ = (0.0. −0.708)	7.15 × 10^−6^
16.	+	+	37	5000	PCA-X	0.827		0.689		
					OPLS-DA	0.897	0.989	0.928	R^2^ = (0.0. 0.704) Q^2^ = (0.0. −0.569)	6.10 × 10^−6^
*The influence of not taking QC into account on statistical models*										
17.	−	−	30	1000	PCA-X	0.748		0.674		
					OPLS-DA	0.657	0.961	0.917	R^2^ = (0.0. 0.294) Q^2^ = (0.0. −0.447)	6.24 × 10^−8^
18.	−	−	37	1000	PCA-X	0.675		0.393		
					OPLS-DA	0.641	0.954	0.903	R^2^ = (0.0. 0.326) Q^2^ = (0.0. −0.477)	1.93 × 10^−7^
19.	−	+	30	1000	PCA-X	0.755		0.682		
					OPLS-DA	0.949	0.999	0.951	R^2^ = (0.0. 0.943) Q^2^ = (0.0. −0.789)	1.04 × 10^−4^
20.	−	+	37	1000	PCA-X	0.763		0.696		
					OPLS-DA	0.819	0.961	0.902	R^2^ = (0.0. 0.285) Q^2^ = (0.0. −0.44)	2.16 × 10^−7^

^1^ CV-ANOVA; ^2^ QC (+/−): QC batch correction included (+) / QC batch correction not included (−); ^3^ Ion mode (+/−): positive ionization mode, ESI+ (+) / negative ionization mode, ESI− (−).

**Table 2:** PCA and OPLS-DA models created using different scaling methods with a 37 min gradient, 5000 counts intensity threshold, inclusion of QC, in positive and negative ionization.

Model	Scaling Method	Type	R^2^X (cum)	R^2^Y (cum)	Q^2^ (cum)
*Positive ionization*					
1.	Centering	PCA-X	0.827		0.689
		OPLS-DA	0.897	0.989	0.928
2.	No-Scaling	PCA-X	0.928		0.904
		OPLS-DA	0.963	0.981	0.909
3.	Unit Variance	PCA-X	0.542		0.380
		OPLS-DA	0.615	0.992	0.929
4.	Unit Variance Normalization	PCA-X	0.716		0.614
		OPLS-DA	0.820	0.991	0.864
5.	Pareto	PCA-X	0.651		0.514
		OPLS-DA	0.589	0.987	0.901
6.	Pareto Normalization	PCA-X	0.815		0.762
		OPLS-DA	0.814	0.892	0.850
*Negative ionization*					
7.	Centering	PCA-X	0.685		0.400
		OPLS-DA	0.657	0.957	0.914
8.	No-Scaling	PCA-X	0.840		0.760
		OPLS-DA	0.888	0.965	0.919
9.	Unit Variance	PCA-X	0.543		0.268
		OPLS-DA	0.494	0.973	0.877
10.	Unit Variance Normalization	PCA-X	0.689		0.545
		OPLS-DA	0.815	0.982	0.882
11.	Pareto	PCA-X	0.593		0.348
		OPLS-DA	0.711	0.995	0.932
12.	Pareto Normalization	PCA-X	0.756		0.690
		OPLS-DA	0.863	0.985	0.915

**Table 3:** The most relevant potential marker compounds related to the increased antioxidant activity identified in buckwheat honey using LC-MS recorded in ESI− and ESI+.

No	Molecular Weight (Da)	Retention Time (s/min)	Compound/Feature
*Negative ionization*			
1	196.0586	0.92	Gluconic acid ^1,2^
2	134.0217	1.05	Malic acid ^1,2^
3	756.2525	1.22	X
4	192.0272	1.42	Citric acid ^1,2^
5	129.0428	1.53	Pyroglutamic acid ^1^
6	291.0955	1.57	N-fructosyl-pyroglutamate ^1^
7	180.0635	1.74	D-Tagatose ^1,^*
8	311.1585	1.87	X
9	181.0703	1.96	Tyrosine ^1^
10	361.1372	1.97	Tyrosine [2M]
11	425.1441	2.02	X
12	343.1268	2.1	N-fructosyl-Tyr ^1^
13	189.1004	2.36	N-Lactoylvaline ^1,^*
14	293.1479	2.41	N-fructosyl-Ile ^1^
15	455.2009	3.44	N-fructosyl-Ile/Leu-hexoside ^1^
16	506.2003	10.11	Linden glycoside isomer I ^1^
17	552.2063	10.52	Linden glycoside isomer II ^1^ [M + HCOOH]
18	506.2003	10.68	Linden glycoside isomer III ^1^
19	1058.404	10.68	Linden glycoside isomer III ^1^ [2M + HCCOH]
20	554.2216	11.09	X
21	166.0632	12.15	Phenyllactic acid ^1,2^
22	200.105	16.55	X
23	202.1205	17.06	Sebacic acid ^1^
*Positive ionization*			
1	115.0637	0.92	L-Proline ^1^
2	277.1159	1.05	N-fructosyl-Pro ^1^
3	459.1946	1.1	X
4	117.0792	1.1	Valine ^1^
5	279.1319	1.23	N-fructosyl-Val ^1^
6	309.1424	1.32	Glucopyranosylfagomine ^1,^*
7	106.0421	1.40	Benzaldehyde ^1^
8	285.1210	1.67	X
9	131.0950	1.77	L-Isoleucine ^1^
10	131.0950	1.88	L-Leucine ^1^
11	122.0371	1.97	Salicylaldehyde ^1^
12	135.0686	1.98	Tyrosine^1^ [M-HCOOH]
13	164.0479	1.98	3′,4′-(methylenedioxy)aceto-phenone ^1^
14	343.1269	2.11	N-fructosyl-Tyr ^1^
15	293.1479	2.29	N-fructosyl-Ile ^1^
16	293.1478	2.43	N-fructosyl-Leu ^1^
17	455.2000	3.44	N-fructosyl-Ile/Leu hexoside ^1^
18	119.0738	3.67	Indoline ^1,^*
19	327.1319	4.20	N-fructosyl-Phe ^1^
20	145.0522	8.17	4-hydroxyquinoline ^1,2^

^1^ Identification by accurate molecular weight, RT, UV spectra and MS^2^ fragmentation analysis based on literature and/or MS^2^ databases; ^2^ Identification confirmed by comparison with analytical standard; * Identified tentatively; X—Not identified.

**Table 4:** Total phenolic content (TPC), reducing power (FRAP) and radical scavenging activity (DPPH) of the analyzed honeys. Data are a mean ± standard deviation (n = 10).

Honey Variety	TPC (mg GAE/kg)	FRAP (mmol Fe^2+^/kg)	DPPH (mg GAE/kg)
Buckwheat	982.63 ± 258.41	6.26 ± 1.96	60.58 ± 25.74
Canola	116.62 ± 24.32	1.34 ± 0.13	11.81 ± 7.39

**Table 5:** Pearson correlation coefficients of correlations between the content of metabolites identified as potential markers of antioxidant activity and phenolic content, antioxidant activity assessed by TPC, DPPH, and FRAP tests.

Compound/Feature	TPC	DPPH	FRAP
*Negative ionization*			
Gluconic acid	**0.667**	**0.540**	**0.636**
Malic acid	0.193	0.145	0.273
756.25247 Da 72.92 s	0.132	−0.020	0.065
Citric acid	**0.677**	**0.525**	**0.646**
Pyroglutamic acid	**0.938**	**0.899**	**0.876**
N-fructosyl-pyroglutamate	**0.926**	**0.933**	**0.931**
D-Tagatose	**0.523**	**0.458**	**0.454**
311.15849 Da 112.26 s	**0.851**	**0.705**	**0.712**
Tyrosine	**0.903**	**0.772**	**0.784**
Tyrosine [2M]	**0.898**	**0.755**	**0.774**
425.14408 Da 121.37 s	**0.893**	**0.763**	**0.793**
N-fructosyl-Tyr	**0.984**	**0.957**	**0.964**
N-Lactoylvaline	**0.887**	**0.896**	**0.905**
N-fructosyl-Ile	**0.920**	**0.956**	**0.953**
N-fructosyl-Ile/Leu-hexoside	**0.975**	**0.928**	**0.935**
Linden glycoside isomer I	**0.678**	**0.699**	**0.671**
Linden glycoside isomer II [M + HCOOH]	**0.668**	**0.693**	**0.661**
Linden glycoside isomer III	**0.666**	**0.703**	**0.627**
Linden glycoside isomer III [2M + HCCOH]	**0.538**	**0.572**	**0.512**
554.22159 Da 665.46 s	**0.607**	**0.651**	**0.579**
Phenyllactic acid	**0.489**	0.421	0.436
554.22159 Da 665.46 s	**0.654**	**0.509**	**0.573**
Sebacic acid	**0.608**	**0.464**	**0.535**
*Positive ionization*			
L-Proline	**0.740**	**0.664**	**0.724**
N-fructosyl-Pro	**0.849**	**0.849**	**0.896**
459.19456 Da 66.29 s	**0.940**	**0.860**	**0.845**
Valine	**0.890**	**0.767**	**0.769**
N-fructosyl-Val	**0.970**	**0.970**	**0.982**
Glucopyranosylfagomine	**0.956**	**0.861**	**0.870**
Benzaldehyde	**0.858**	**0.703**	**0.731**
285.12095 Da 99.98 s	**0.981**	**0.926**	**0.955**
L-Isoleucine	**0.935**	**0.819**	**0.840**
L-Leucine	**0.929**	**0.821**	**0.835**
Salicylaldehyde	**0.939**	**0.821**	**0.846**
Tyrosine [M-HCOOH]	**0.938**	**0.822**	**0.843**
3′,4′-(methylenedioxy)aceto-phenone	**0.939**	**0.826**	**0.842**
N-fructosyl-Tyr	**0.965**	**0.969**	**0.978**
N-fructosyl-Ile	**0.961**	**0.965**	**0.976**
N-fructosyl-Leu	**0.966**	**0.964**	**0.977**
N-fructosyl-Ile/Leu hex	**0.974**	**0.933**	**0.952**
Indoline	**0.615**	**0.530**	**0.538**
N-fructosyl-Phe	**0.843**	**0.843**	**0.849**
4-hydroxyquinoline	**0.814**	**0.831**	**0.836**

Positive and statistically significant correlation (*p* < 0.05) are marked in bold.

## Data Availability

The data presented in this study are available on request from the corresponding author.

## Supplementary Materials

The following supporting information can be downloaded at: https://www.mdpi.com/article/10.3390/molecules30102198/s1, Figure S1: Examples of correlations between the content of metabolites identified as potential markers of antioxidant activity and antioxidant activity (DPPH and FRAP tests).

## Abbreviations

The following abbreviations are used in this manuscript:

LC-MS	Liquid Chromatography–Mass Spectrometry
PCA	Principal Component Analysis
OPLS-DA	Orthogonal Projections to Latent Structures Discriminant Analysis
QC	Quality Control
TPC	Total Phenolic Content
DPPH	2,2-Diphenyl-1-picrylhydrazyl (radical scavenging assay)
FRAP	Ferric Reducing Antioxidant Power
RT	Retention Time
ESI+/ESI−	Electrospray Ionization Positive / Negative Mode
MS/MS	Tandem Mass Spectrometry
UV	Ultraviolet (Spectroscopy)
GAE	Gallic Acid Equivalent
