# Supplementary material for: Application of Liquid Chromatography–Mass Spectrometry-Based Untargeted Metabolomics to Reveal Metabolites Related to Antioxidant Activity in Buckwheat Honey
Source: Molecules. 2025 May 17;30(10):2198. doi: 10.3390/molecules30102198 (PMC12114437; doi:10.3390/molecules30102198)
Supplement: Supplementary file 1 [file molecules-30-02198-s001.zip › molecules-3642572-supplementary.pdf]

Supplementary Materials

# Application of Liquid Chromatography–Mass Spectrometry-Based Untargeted Metabolomics To Reveal Metabolites Related to Antioxidant Activity in Buckwheat Honey

**Emilia Pogoda † and Piotr M. Kuś ***

Department of Pharmacognosy and Herbal Medicines, Faculty of Pharmacy, Wroclaw Medical University, ul. Borowska 211a, 50-556 Wrocław, Poland; † student.

* Correspondence: e-mail address: piotr.kus@umw.edu.pl

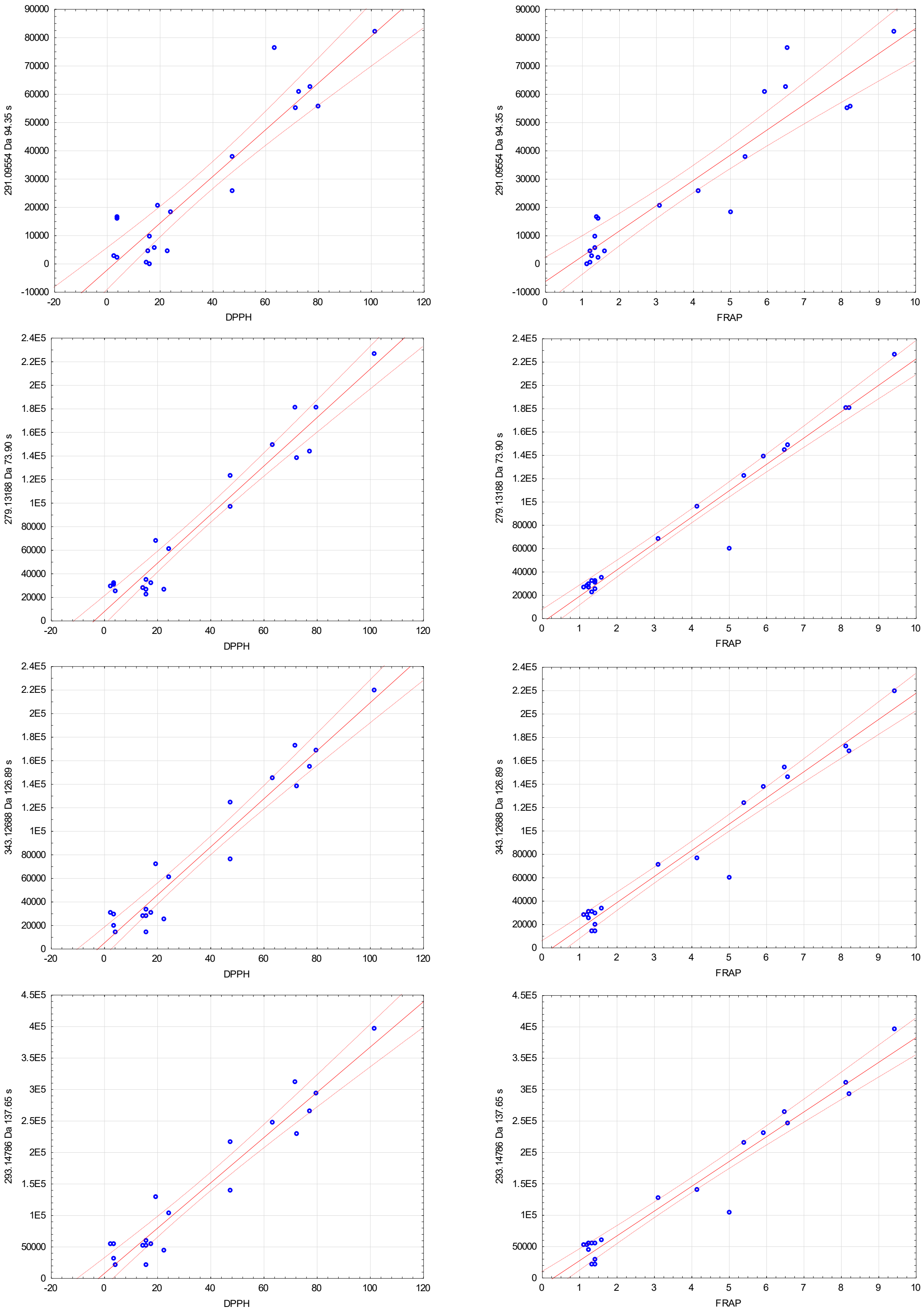


**Figure S1.** Examples of correlations between the content of metabolites identified as potential markers of antioxidant activity and antioxidant activity (DPPH and FRAP tests)
